# A case report of transfusion-transmitted Plasmodium malariae from an asymptomatic non-immune traveller

**DOI:** 10.1186/1475-2875-12-439

**Published:** 2013-12-05

**Authors:** Emmaline E Brouwer, Jaap J van Hellemond, Perry JJ van Genderen, Ed Slot, Lisette van Lieshout, Leo G Visser, Pieter J Wismans

**Affiliations:** 1 Department of Internal Medicine, Harbour Hospital and Institute for Tropical Diseases, Haringvliet 2 3011 TD, Rotterdam, The Netherlands; 2 Department of Medical Microbiology and Infectious Diseases, Erasmus University Medical Centre and Rotterdam Harbour Hospital and Institute for Tropical Diseases, Rotterdam, The Netherlands; 3 Department of Medical Affairs, Sanquin Blood Supply Foundation, Amsterdam, The Netherlands; 4 Department of Parasitology and Department of Medical Microbiology, Leiden University Medical Centre, Leiden, The Netherlands; 5 Department of Infectious Diseases, Leiden University Medical Centre, Leiden, The Netherlands

**Keywords:** Malaria, Blood transfusion, Blood safety, Blood donor screening, *Plasmodium malariae*, Transfusion-transmitted malaria, Blood-borne, Infection, Thrombocytopenia, Asymptomatic malaria, Look-back

## Abstract

**Background:**

The incidence of transfusion-transmitted malaria is very low in non-endemic countries due to strict donor selection. The optimal strategy to mitigate the risk of transfusion-transmitted malaria in non-endemic countries without unnecessary exclusion of blood donations is, however, still debated and asymptomatic carriers of *Plasmodium* species may still be qualified to donate blood for transfusion purposes.

**Case description:**

In April 2011, a 59-year-old Dutch woman with spiking fevers for four days was diagnosed with a *Plasmodium malariae* infection. The patient had never been abroad, but nine weeks before, she had received red blood cell transfusion for anaemia. The presumptive diagnosis of transfusion-transmitted quartan malaria was made and subsequently confirmed by retrospective PCR analysis of donor blood samples. The donor was a 36-year-old Dutch male who started donating blood in May 2006. His travel history outside Europe included a trip to Kenya, Tanzania and Zanzibar in 2005, to Thailand in 2006 and to Costa Rica in 2007. He only used malaria prophylaxis during his travel to Africa. The donor did not show any abnormalities upon physical examination in 2011, while laboratory examination demonstrated a thrombocytopenia of 126 × 10^9^/L as the sole abnormal finding since 2007. Thick blood smear analysis and the *Plasmodium* PCR confirmed an ongoing subclinical *P. malariae* infection. Chloroquine therapy was started, after which the infection cleared and thrombocyte count normalized. Fourteen other recipients who received red blood cells from the involved donor were traced. None of them developed malaria symptoms.

**Discussion:**

This case demonstrates that *P. malariae* infections in non-immune travellers may occur without symptoms and persist subclinically for years. In addition, this case shows that these infections pose a threat to transfusion safety when subclinically infected persons donate blood after their return in a non-endemic malaria region.

Since thrombocytopenia was the only abnormality associated with the subclinical malaria infection in the donor, this case illustrates that an unexplained low platelet count after a visit to malaria-endemic countries may be an indicator for asymptomatic malaria even when caused by non-falciparum *Plasmodium* species.

## Background

Transfusion-transmitted malaria (TTM) was first described in 1911 [1]. The most recent publication on global incidence of TTM, based on data from 1911 to 1979 [2], suggests that the incidence of TTM is about 145 reported cases per year, mostly confined to endemic countries. The relatively high likelihood of TTM via donor blood in sub-Saharan African countries is illustrated by a median prevalence of malaria, determined by microscopic evaluation of thick blood smears, of 10.2% (range: 0.7% in Kenya to 55% in Nigeria) in donor blood samples [3]. In endemic countries differentiating cases of TTM from natural infections is a challenge as malaria, occurring post-transfusion, can be the result of either a natural infection or transfusion-transmitted. Hence, the number of TTM in endemic countries is unquestionably under-reported. In striking contrast, in non-endemic countries the incidence of TTM is low, due to strict donor selection. Three cases were reported in Canada between 1994 and 1999 [4], 14 in the USA between 1990 and 1999 [5] and two in the UK since 1996 [6]. In the past decade, two cases were reported from other European countries, both with fatal consequences for the recipient [7,8].

The optimal strategy to minimize the risk of TTM in non-endemic countries without unnecessary exclusion of blood donations is still a matter of debate. Reesink and colleagues provided an excellent overview of current strategies in a number of (mainly European) non-endemic countries [9,10]. In short, most countries apply a strict donor deferral system based on travel history. However, this strategy is not optimal because many healthy donors are deferred unnecessarily, leading to donation loss, and lengthy deferrals may discourage donors to return at all [1]. Despite these strict donor deferral systems, some asymptomatic carriers of *Plasmodium* spp. may still be accepted for blood donation, and therefore the possibility of TTM is not completely excluded ([11,12] and this case report).

Potential donor exposure to acquisition of malaria parasites is an increasing problem due to the substantial rise in global travelling and immigration. Therefore, it is more challenging than ever to ensure that the blood supply in non-endemic areas is devoid of potential malaria infections. Donor selection measures, such as geographical-risk questions in order to identify the donors at risk and to temporarily defer them, have been implemented by the blood bank community. In addition, some blood transfusion services in non-endemic areas implemented laboratory testing for shortened deferrals and/or to further reduce the risk of TTM. Donors can be tested by thick blood smear examination, malarial antibody testing and *Plasmodium* DNA detection by PCR [1,13,14]. It is widely accepted that none of these strategies is perfect, due to either lack of test sensitivity or unfavorable cost efficiency. The optimal approach for a given location will vary according to the background level of malaria risk faced by the donor and recipient population, in combination with the resources available.

## Case presentation

A case is presented of transfusion-transmitted *Plasmodium malariae* infection from a long-term asymptomatic, non-immune traveller who had not experienced any clinical malaria before.

### The recipient

A 59-year-old Dutch woman was seen at the outpatient clinic of the Leiden University Medical Centre (LUMC) on April 14^th^ 2011, because of spiking fevers for four days. Two months before, on February 14^th^, she underwent coronary-artery bypass surgery because of symptomatic coronary artery disease (Table 1). She had been feeling tired and nauseous and suffered from a non-productive cough and chest pain on deep inspiration since. She presented with daily fevers up to 38.8°C accompanied by chills, headache, nausea, and night sweats. Upon physical examination no abnormalities were found. Laboratory tests revealed leukocytopaenia (3.6 × 10^9^/L) and thrombocytopenia (71 × 10^9^/L). In the peripheral blood smear malaria parasites were seen which were subsequently identified as *P. malariae* by morphology and PCR. The parasitaemia was 2%. The patient was treated with chloroquine (25 mg/kg) and recovered completely.

**Table 1:** Overview of most relevant events

**Date**	**Donor/Recipient**	**Event**
August 2005	Donor	Visit to Kenya, Tanzania and Zanzibar
3 April 2006	Donor	First visit to blood bank for examination and giving blood samples for testing
4 May 2006	Donor	Blood donation #1
2 August 2006	Donor	Blood donation #2
Aug – Sept 2006	Donor	Visit to Thailand (only to low risk areas for malaria, no precautions required)
9 November 2006	Donor	Blood donation #3
27 February 2007	Donor	Blood donation #4 at a thrombocyte count of 141 × 10^9^/L
22 May 2007	Donor	Blood donation #5 at a thrombocyte count of 94 × 10^9^/L
29 May 2007	Donor	Informed about thrombocytopenia; referred to GP
14 August 2007	Donor	Blood donation #6
September 2007	Donor	Visit to Costa Rica
26 May 2008	Donor	Blood donation #7
25 August 2008	Donor	Blood donation #8
17 November 2008	Donor	Blood donation #9
11 February 2009	Donor	Blood donation #10
8 June 2009	Donor	Blood donation #11
3 December 2009	Donor	Blood donation #12
1 March 2010	Donor	Blood donation #13
10 May 2010	Donor	Blood donation #14
9 February 2011	Donor	Blood donation #15
14 February 2011	Recipient	Coronary-artery bypass surgery and transfusion of red blood cell concentrate from blood donation #15
14 April 2011	Recipient	Diagnosis *P. malariae* infection
14 April 2011	Donor	Informed about post-transfusion malaria infection in recipient
7 June 2011	Donor	Blood collection for malaria tests
23 June 2011	Donor	First laboratory evidence for subclinical *P. malariae* infection in specimen collected on 7 June 2011
17 August 2011	Donor	First visit to Harbour hospital and initial examination
8 November 2011	Donor	Second visit to Harbour hospital; confirmation of on-going, subclinical *P. malariae* infection
10 November 2011	Donor	Start oral chloroquine treatment

The patient had never been abroad, neither had she recently been near an international airport. During her admission in February 2011, she had received one unit of packed red blood cells immediately after surgery. Hence, the presumptive diagnosis of transfusion-transmitted quartan malaria was made.

### The donor

The Dutch blood bank was notified immediately about the presumptive diagnosis of TTM, after which the involved donor was informed and donation records were retrieved. Stored plasma from the involved donation as well as subsequent collected blood was examined for malaria (Tables 1 and 2). Thin and thick blood smears were negative, but immune fluorescence assay (IFA) was weakly positive and the sensitive *Plasmodium-*specific semi-quantitative real-time PCR showed a weak, though positive signal for *P. malariae* (Ct-value 38), confirming the persistent, asymptomatic *P. malariae* infection in the donor (Table 2).

**Table 2:** Overview of relevant laboratory test results of donor

**Collection date**	**Laboratory test**	**Result**	**Specimen**
27 February 2007	Thrombocyte count	141 × 10^9^/L	Fresh blood
22 May 2007	Thrombocyte count	94 × 10^9^/L	Fresh blood
9 February 2011	*Plasmodium* species PCR *	Negative	Stored plasma
	Malaria serology by ELISA *	Negative (0.36, cut off <1.0)	Stored plasma
	Malaria serology by immunofluorescence assay (IFA) *	Positive (1:40, cut-off <1:40)	Stored plasma
7 June 2011	*Plasmodium* genus PCR	Positive for *Plasmodium* spp.	EDTA-blood
	Malaria serology by immunofluorescence assay (IFA)	Positive (1:160, cut-off <1:40)	Serum
	*Plasmodium* species PCR	Positive for *P. malariae*	EDTA-blood
17 August 2011	Thrombocyte count	126 × 10^9^/L	Fresh blood
	Thick blood smear, QBC, malaria antigen test	Negative	Fresh blood
8 November 2011	Thrombocyte count	128 × 10^9^/L	Fresh blood
	QBC, malaria antigen test, thin blood smear, malaria serology (ELISA)	Negative	Fresh blood
	Thick blood smear	One structure suspected for *P. malariae*	Fresh blood
	*Plasmodium* species PCR	Positive for *P. malariae*	Fresh blood
	*Plasmodium* species PCR	Positive for *P. malariae*	Bone marrow biopsy
	*Leishmania* spp. PCR	Negative	Bone marrow biopsy
26 November 2011	Thrombocyte count	144 × 10^9^/L	Fresh blood
1 December 2011	Thrombocyte count	157 × 10^9^/L	Fresh blood
12 July 2012	Thrombocyte count	139 × 10^9^/L	Fresh blood
	Thick and thin blood smears, QBC, malaria antigen test, malaria serology (ELISA), *Plasmodium* species PCR	Negative	Fresh blood
6 March 2013	Thrombocyte count	209 × 10^9^/L	Fresh blood
	Thick and thin blood smears, QBC, malaria antigen test, malaria serology (ELISA), *Plasmodium* species PCR	Negative	Fresh blood

The tests were performed in the following laboratories: Diagnostic Services, Sanquin Blood Supply Foundation, Amsterdam (2007 and malaria serology by ELISA first half 2011); Dept. Medical Microbiology, University Medical Centre St Radboud, Nijmegen, and/or Dept. Parasitology, Leiden University Medical Centre, Leiden (first half 2011); the Harbour Hospital Laboratory, Dept. Medical Microbiology and Infectious Diseases and Clinical Chemistry, Erasmus University Medical Centre and Harbour Hospital, Rotterdam (from August 2011 onwards). Tests marked by an *indicate results of tests performed retrospectively in May to July 2011 on materials collected on 9 February 2011.

**Figure 1:**
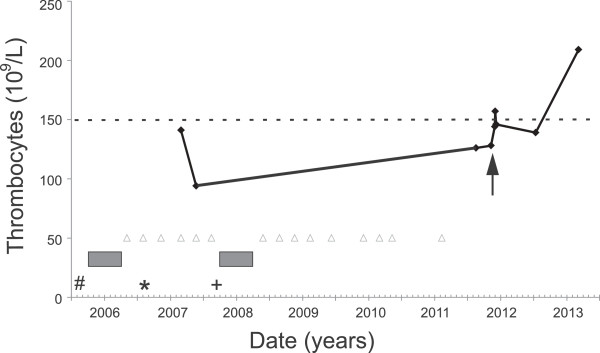
**Time line of important donor events.** Blood donations by the donor are indicated by open triangles. Visits to Africa, Thailand and Costa Rica are indicated by #, * and +, respectively. Deferral periods are indicated by grey bars. The solid line connects the platelet count numbers in course of time. The dashed line indicates the lower reference value for normal thrombocyte concentrations. The arrow indicates the start of oral chloroquine treatment.

The blood donor was then referred to the Rotterdam Harbour Hospital in August 2011. The donor was a 36-year-old Dutch male without relevant medical history. In particular, he did not have any complaints of fever or chills in the past five years, nor did he take any medication. The donor had a secretarial job and lived about 50 km from the nearest international airport. He started donating blood in May 2006 and had given 15 whole blood donations until 2011 (Table 1). His travel history outside Europe included a trip to Kenya, Tanzania and Zanzibar in 2005, for which he had used atovaquone/proguanil as malaria prophylaxis. In 2006 and 2007 he visited Thailand and Costa Rica, respectively. On both occasions he did not use malaria chemoprophylaxis. According to the donor, he only stayed in low risk areas for malaria in Thailand. Therefore, the donor was only deferred from blood donations for six months following his visit to Africa and Costa Rica, according to the European Union directive (Figure 1) [15].

**Figure 2:**
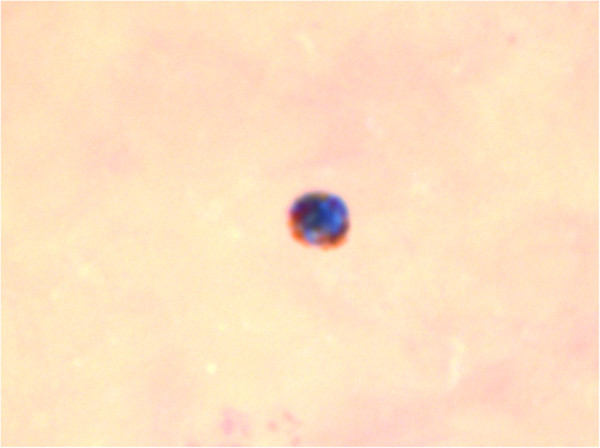
Giemsa-stained thick blood smear from the donor with a structure suspected for a malaria parasite.

Physical examination showed no abnormalities and the spleen was not enlarged (11.5 cm) on abdominal ultrasound examination. Laboratory examination demonstrated a thrombocytopenia of 126 × 10^9^/L, hemoglobin 9.6 mmol/L, leucocyte count of 4.1 × 10^9^/L and lactate dehydrogenase (LDH) 163 U/L. Standard malaria diagnostics by a malaria rapid test, thin and thick blood smears as well as quantitative buffy coat (QBC) analysis were negative. However, upon his second visit in November 2011, a single malaria parasite was detected after meticulous investigations of over five thick and five thin blood smears (Figure 2). A subsequently ordered *Plasmodium-*specific semi-quantitative real-time PCR was positive for *P. malariae* (Ct-value 37), confirming an ongoing subclinical *P. malariae* infection. The high Ct-values (37-38) observed in the subsequent blood specimens of the donor are close to the detection level of these tests and correspond to extremely low parasite numbers in the order of 1-100 parasites per mL.

Plasma and thrombocytes from the donor were never used for transfusion, but red blood cells were transfused into 15 recipients. None of the other 14 recipients developed malaria symptoms after transfusion. When the look-back procedure was performed, already 11 recipients had died of reasons other than malaria. The remaining three recipients were invited for a thorough malaria screening. One recipient was tested negative for malaria by both serology and real-time PCR. The two other recipients had no clinical signs of malaria and declined the invitation.

In retrospect, laboratory screening by the blood bank upon application of the donor to volunteer for platelet donations by aphaeresis, already revealed an unexplained thrombocytopenia (94 × 10^9^/L) in February and May 2007. The blood bank staff then advised the donor to consult his general practitioner. To exclude other causes of thrombocytopenia apart from malaria, bone marrow morphology was examined, which demonstrated no abnormalities. In addition, no platelet antibodies were detected and the donor had normal thrombopoietin levels (13 E/mL; n = 4-32). All results together with the longstanding thrombocytopenia and travel history demonstrated that the donor must have had a subclinical *P. malariae* infection for at least four years and that the infection was most probably acquired during his travel through Kenya, Tanzania and Zanzibar in 2005 or during his visit to Thailand in 2006. He was treated orally with chloroquine 600 mg followed by a dose of 300 mg after six, 24 and 48 hours, respectively. On follow-up, his platelet count quickly normalized (Figure 2) and all malaria tests (thick and thin blood smears, QBC, malaria rapid tests, PCR, and serology) turned negative (Table 2).

## Discussion

This case of TTM is remarkable, not only for being the first TTM case in the Netherlands in 43 years [16], but also because the donor was a non-immune traveller from a non-endemic country who never suffered from malaria and was without any symptoms for at least four years after acquisition of the *P. malariae* infection. Parasite counts in *P. malariae* are usually low, due to the low number of merozoites produced per asexual cycle in the erythrocyte, the slow replication cycle of 72 hours in erythrocytes and the preference of the parasite to develop in older erythrocytes. These factors usually allow for earlier development of immunity by the human host if left untreated. Blood stage *P. malariae* can persist for extremely long periods, often, it is believed, for the life of the human host [17]. Although subclinical malaria is relatively common in endemic countries [18], asymptomatic *P. malariae* infections in non-immune travellers are probably very rare, as reports of experimental *P. malariae* infections for the treatment of neurosyphilis patients between 1940 and 1963 recorded only a single patient without fever out of 69 infected patients [17,19].

Thrombocytopenia is a common feature of malaria, occurring in 24-93% of cases of acute disease [20,21]. In asymptomatic *Plasmodium* spp. carriers thrombocyte numbers are unknown, although one paper from Nigeria showed that asymptomatic carriers of *Plasmodium* spp. had a moderately lower platelet count than non-parasitaemic subjects [22]. The only abnormality in the donor in this case was a thrombocytopenia that was first observed in May 2007 and persisted until the malaria infection was cleared by chloroquine treatment. This now suggests that thrombocytopenia may be an indicator for asymptomatic infections by *Plasmodium* species other than *P. falciparum* as well. Further studies are needed to determine whether, and to what extent, this finding holds true in a larger group of asymptomatic carriers of *Plasmodium* spp.

For asymptomatic visitors to malaria-endemic areas, other than ex-residents and individuals with a history of malaria infection, the European Union directive for technical requirements for blood and blood components states that they shall be excluded from blood donation for six months after leaving the endemic area [15]. The donor in this case was qualified to donate blood according with the provisions of this directive (Table 1). This illustrates that a donor deferral policy based on geographic-risks reduces the risks of TTM, but cannot prevent incidents from occurring. It is noteworthy that the most commonly used serological test for screening donors for IgG antibodies to *Plasmodium* spp., the Malaria Total Antibody EIA kit (Lab21, Healthcare Ltd., Kentford, UK), used by the Dutch blood bank as well as by several other blood transfusion services in non-endemic countries to shorten deferral periods for ex-residents of malaria endemic areas and for individuals with a history of malaria, did not detect this asymptomatic malaria infection in the donor. This test was performed retrospectively (Table 2).

Only expensive methods, such as the sensitive serological immune fluorescence assay (IFA) and real-time PCR analysis (Table 2), did detect the asymptomatic *P. malariae* infection in the donor. As long as effective methods for pathogen inactivation of red cell units or whole blood are not available, cases of TTM can continue to occur in areas that are not endemic for malaria, irrespective of the safety measures currently adopted by the blood bank community.

## Conclusion

This case demonstrates that asymptomatic chronic *P. malariae* infections can occur in non-immune persons after a visit to malaria-endemic areas and thus pose a continued threat to transfusion safety. Since thrombocytopenia was the only abnormality associated with this asymptomatic *P. malariae* infection in the donor, an unexplained low platelet count may be an indicator for asymptomatic malaria even when caused by non-falciparum *Plasmodium* species.

### Consent

Written informed consent was obtained from the patients for publication of this case report and any accompanying images. A copy of the written consent is available for review by the Editor-in-Chief of this journal.

## Competing interests

The authors declare that they have no competing interests.

## Authors’ contributions

EEB, JvH, PG, LVL, ES, LV and PJW contributed to acquisition of the clinical and laboratory data. EEB prepared the draft version of manuscript and all authors contributed to the concept and design of manuscript and approved its final version.
